# mSphere of Influence: Systemic decoding gene function in *Toxoplasma gondii* pathogenesis—CRISPR screens and beyond

**DOI:** 10.1128/msphere.00271-23

**Published:** 2023-06-29

**Authors:** Yifan Wang

**Affiliations:** 1 Department of Microbiology and Immunology, University of Michigan Medical School, Ann Arbor, Michigan, USA; University at Buffalo, Buffalo, New York, USA

**Keywords:** *Toxoplasma gondii*, CRISPR screen, functional genomics

## Abstract

Yifan Wang works in the field of molecular parasitology with a focus on host-pathogen interactions. In this mSphere of Influence article, he reflects on how papers entitled “A genome-wide CRISPR screen in *Toxoplasma* identifies essential apicomplexan genes” by S. M. Sidik, D. Huet, S. M. Ganesan, M.-H. Huynh, et al. (Cell 166:1423.e12–1435.e12, 2016, https://doi.org/10.1016/j.cell.2016.08.019) and “Mapping host-microbe transcriptional interactions by dual Perturb-seq” by S. Butterworth, K. Kordova, S. Chandrasekaran, K. K. Thomas, et al. (bioRxiv, https://doi.org/10.1101/2023.04.21.537779) made an impact on his research and changed the way he thinks how functional genomics and high-throughput screens provide novel insights into pathogen pathogenesis.

## TEXT

*Toxoplasma gondii* is an obligate intracellular parasite that causes severe disease in fetuses and immunocompromised individuals (1). The parasite can invade all the nucleated cells and establish infection in almost all warm-blooded animals including humans, which is estimated that one-third of the human population is chronically infected with *Toxoplasma*. In addition to its public health significance, *Toxoplasma* is experimentally tractable and has emerged as a model to study the biology of other apicomplexan parasites of medical and veterinary importance (2). However, considering that a large portion of predicted proteins lacks a defined role in the biology of *Toxoplasma*, the need for a systemic analysis of all parasite protein-coding genes has emerged as an immediate need in the field. You can therefore imagine my excitement on reading the papers by Sidik et al. (3), which shows the power of CRISPR/Cas9-mediated screens in unraveling the complex biology of *Toxoplasma*, and by Butterworth et al. (4), which shows that combining CRISPR screens with single-cell RNA sequencing (RNA-seq) provides invaluable insights into *Toxoplasma*-induced host transcriptional modification.

CRISPR technology enables efficient genetic manipulation by utilizing the Cas9 endonuclease and single-guide RNAs (sgRNAs) to introduce targeted double-stranded DNA break in almost any given genomic location. In the paper by Sidik et al. (3), the Lourido lab developed the first CRISPR/Cas9-mediated genome-wide knockout strategy in *Toxoplasma* by transfecting an sgRNA library consisting of sgRNAs targeting all 8,156 *Toxoplasma* protein-coding genes (10 sgRNAs per gene) into the Cas9-expressing parasite. The authors then propagated obtained parasite mutant population in host cells and assessed the overall proliferation of each individual knockout parasite *in vitro*. Typically, a *Toxoplasma* mutant with compromised *in vitro* growth also demonstrated a defect in parasite pathogenicity and virulence *in vivo*. On determining the relative abundance of sgRNAs targeting any given gene within the population using Illumina sequencing, genes exhibiting significant diminishment in sgRNA count over time were defined as those conferring fitness to *Toxoplasma*. The results were insightful as this study discovered a wealth of previously unidentified genes essential for the proliferation of *Toxoplasma*, many of which are conserved across the Apicomplexa phylum. This is particularly exciting as it provides novel insights into shared genetic dependencies in related parasites such as *Plasmodium* spp., the causative agents of malaria. In the end, the authors demonstrated that one of the identified genes is critical for *Toxoplasma* to invade host cells, and its ortholog also plays an essential role in the asexual cycle of *Plasmodium falciparum*.

This study is a groundbreaking contribution to the field of molecular parasitology. The data generated from this paper serve as a precious genetic asset for researchers to unravel the genes associated with *Toxoplasma* pathogenesis. A “phenotype” score assigned to each gene based on the change in abundance for its sgRNAs from the CRISPR screen was incorporated into ToxoDB (an online integrated *Toxoplasma* database) and used as an estimation for the essentiality of a particular gene to overall parasite fitness. This paper was inspiring because it facilitates systemic investigations into the biology of *Toxoplasma* and other apicomplexan parasites, thereby energizing the research landscape for the field. The CRISPR/Cas9 screens have been applied in several studies to identify genes involved in various aspects of *Toxoplasma* pathogenesis, including my own research, in which we performed the screen to identify parasite genes that counteract host interferon-gamma response (5). This paper also provides a blueprint for the development of CRISPR/Cas9-based functional genomic techniques in *Toxoplasma*, for example, a conditional splitCas9 phenotypic screen (6) and CRISPR-mediated single-cell analysis (also called Perturb-seq, see below in detail).

In the paper by Butterworth et al. (4), the Treeck lab applied a targeted CRISPR screen coupled with single-cell RNA-seq in parasite-infected cells to understand *Toxoplasma*-secreted effector-mediated host reprogramming at a systemic level. To successfully establish an infection, *Toxoplasma* utilizes its protein secreted from two unique organelles, known as rhoptry and dense granule, to alter the host gene expression, modulate the immune response, and mediate pathogenesis (7). The study focused on 256 genes encoding rhoptry and dense granule proteins and generated a pooled CRISPR knockout population followed by evaluating their role in changing the transcriptional profiling of infected cells. This screen not only validated all previously known parasite proteins that modulate host transcriptional response but also identified novel effectors contributing to multiple steps of *Toxoplasma*’s process of co-opting the host cells. For instance, the authors discovered dense granule protein GRA59, which is involved in the trafficking of other parasite-secreted proteins into the host nucleus to manipulate the host transcriptional process, and TgSOS1, a novel effector essential for subverting the expression of pro-inflammatory genes in macrophages.

This particular paper caught my attention due to its innovative approach and the significant implications it holds for the field of parasitology. This study is the first attempt to map the host-pathogen transcriptional interactions via Perturb-seq. It presents an incredibly powerful tool to screen pathogen effectors associated with host modulation, which propels the field beyond the confines of conventional parasite fitness as primary quantitative phenotypic indicators and broadens the application of CRISPR screens into a more comprehensive investigation of *Toxoplasma* pathogenesis. Furthermore, an in-depth exploration of the data derived from this study has the potential to elucidate critical host genes/pathways that significantly influence the course of *Toxoplasma* infection. Overall, these two pioneering studies underscore the promise of sophisticated functional genomic approaches in unveiling the molecular mechanisms of *Toxoplasma* pathogenesis and provide the scientific framework for developing novel anti-*Toxoplasma* therapeutic strategies.
